# Supplementary material for: Axonal Protection by Oral Nicotinamide Riboside Treatment with Upregulated AMPK Phosphorylation in a Rat Glaucomatous Degeneration Model
Source: Curr Issues Mol Biol. 2023 Aug 25;45(9):7097–109. doi: 10.3390/cimb45090449 (PMC10527704; doi:10.3390/cimb45090449)
Supplement: Supplementary file 1 [file cimb-45-00449-s001.zip › cimb-2539986-supplementary.pdf]

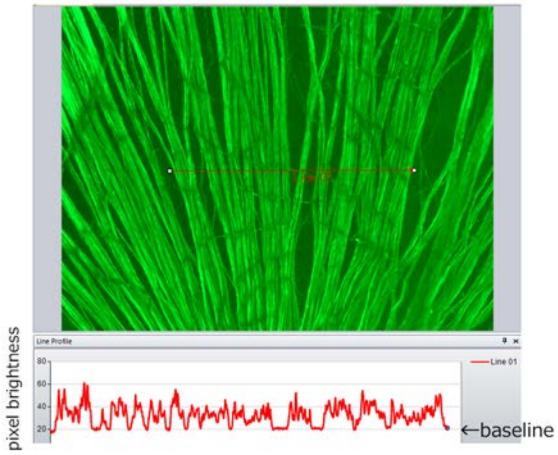

Supplementary Figure S1. The quantitative pixel brightness values were expressed as the area of under curve which means total sum of pixel value in the analysis line length. Red analysis line = 500 μm

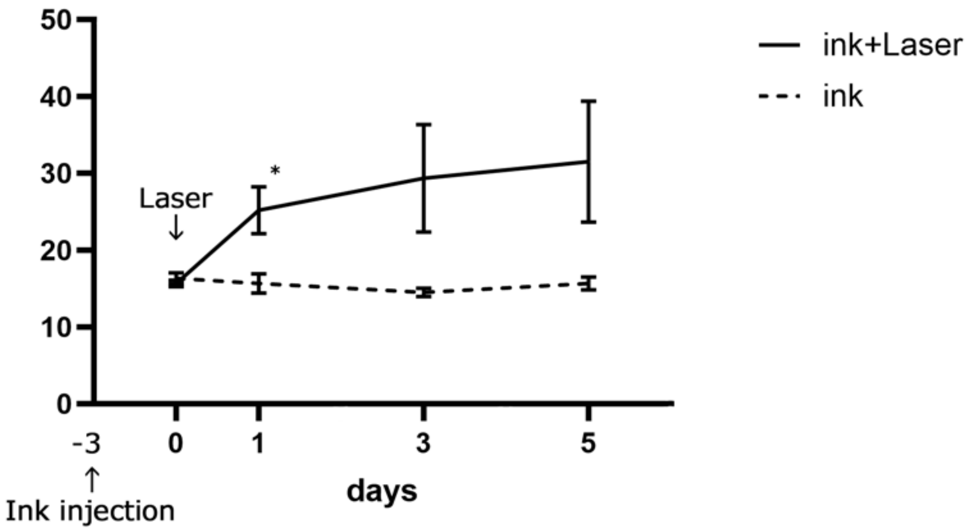

Supplementary Figure S2. Time course of IOP changes in the sham (n = 6) and experimental glaucoma (n = 6) groups. Although IOP tended to be higher in the laser group compared to the no-laser group (sham), statistical significance was observed in only day 1 (p * < 0.05 vs. sham).
